# Cutaneous Secondary Syphilis Resembling Non-Melanoma Skin Cancer

**DOI:** 10.7759/cureus.10774

**Published:** 2020-10-02

**Authors:** Courtney E Stone, Ndidi-Amaka Onyekaba, Matthew Lucas, Drazen Jukic

**Affiliations:** 1 Medicine, Mercer University School of Medicine, Savannah, USA; 2 Dermatology, Winn Army Community Hospital, Fort Stewart, USA; 3 Dermatology, University of Florida, Gainesville, USA; 4 Pathology, Mercer University School of Medicine, Savannah, USA; 5 Dermatopathology, Georgia Dermatopathology, Savannah, USA; 6 Telepathology, James A. Haley Veterans' Hospital, Tampa, USA

**Keywords:** cutaneous syphilis, treponema pallidum, immunohistochemistry staining, non-melanoma skin cancer, secondary syphilis

## Abstract

The cutaneous manifestations of secondary syphilis can vary significantly between patients, leading to a more difficult or delayed diagnosis. Here we present an instructive case of secondary syphilis in a 45-year-old, HIV-positive male patient. He presented with a solitary, crusted anterior neck nodule without concomitant systemic symptoms. Together, history and physical exam were concerning for non-melanoma skin cancer. Histopathologic evaluation of the lesion revealed an extensive infiltrate of plasma cells at the dermoepidermal junction, and immunohistochemical staining revealed numerous *Treponema pallidum* microorganisms. Physicians must keep syphilis in the differential diagnosis when evaluating atypical nodular lesions resembling non-melanoma skin cancer for the purpose of initiating appropriate antibiotic treatment and preventing future infectious complications.

## Introduction

Syphilis is a well-known sexually transmitted infection caused by the bacterium *Treponema pallidum*. The prevalence of primary and secondary syphilis cases in the United States has been steadily rising since the lowest reported rates in 2001 [1]. This rise is presumed to be attributable to an increased number of cases diagnosed in men who have sex with men (MSM) and patients with HIV coinfection [1,2]. Spirochetes initially gain access to the body through microtrauma of skin and mucosa and disseminate via hematogenous spread [3]. The primary presentation of syphilis is a well-described painless chancre at the site of inoculation with concurrent or delayed local lymphadenopathy [3,4]. The chancre appears between one week and three months after initial exposure and can self-resolve. If left untreated, *Treponema* may spread systemically and develop into secondary syphilitic infection weeks to months later [4].

Secondary syphilis has a wide range of systemic manifestations, including constitutional symptoms, rash, lymphadenopathy, and neurologic complications. The classic cutaneous manifestation of secondary syphilis is described as a generalized, copper-colored maculopapular rash involving the palmar and plantar surfaces [3-5]. However, this characteristic appearance is not always present, and atypical cutaneous manifestations vary from nodular solitary lesions to pustular, annular, or framboesiform dermatoses [5,6]. The ambiguous picture of cutaneous secondary syphilis has prompted its nickname, the great imitator, as lesions are commonly mistaken for alternate diagnoses [4,5]. If secondary manifestations of syphilis are not recognized or treated appropriately, spirochetes may enter the latent stage of infection and ultimately develop into tertiary syphilis.

Tertiary syphilis has the potential to cause debilitating harm on the body, involving the cardiovascular and central nervous systems [3,4]. Furthermore, nodular lesions, known as gummae, are quite common in the tertiary presentation of syphilis. In order to stop the progression of infection to the tertiary stage, cases of primary and secondary syphilis must be treated promptly.

## Case presentation

A 45-year-old male goat farmer presented to the dermatology clinic with a pertinent past medical history of cutaneous squamous cell carcinoma and HIV infection. At the time, he reported he was sexually active and monogamous with a male partner. He complained of a firm, erythematous, centrally crusted nodule on the anterior neck, measuring 18x10 mm in size. The lesion appeared three weeks prior to the office visit and was described as enlarging and nonpruritic. The patient denied associated systemic symptoms including fever, headache, and general malaise. A waist up physical exam was performed with no other cutaneous findings other than lentigines and seborrheic keratoses. There was no regional lymphadenopathy, tenderness to palpation, or mucosal involvement appreciated. Differential diagnoses at the time included squamous cell carcinoma versus an infectious process. The Orf virus was considered because of the patient’s occupation as a goat farmer.

**Figure 1:**
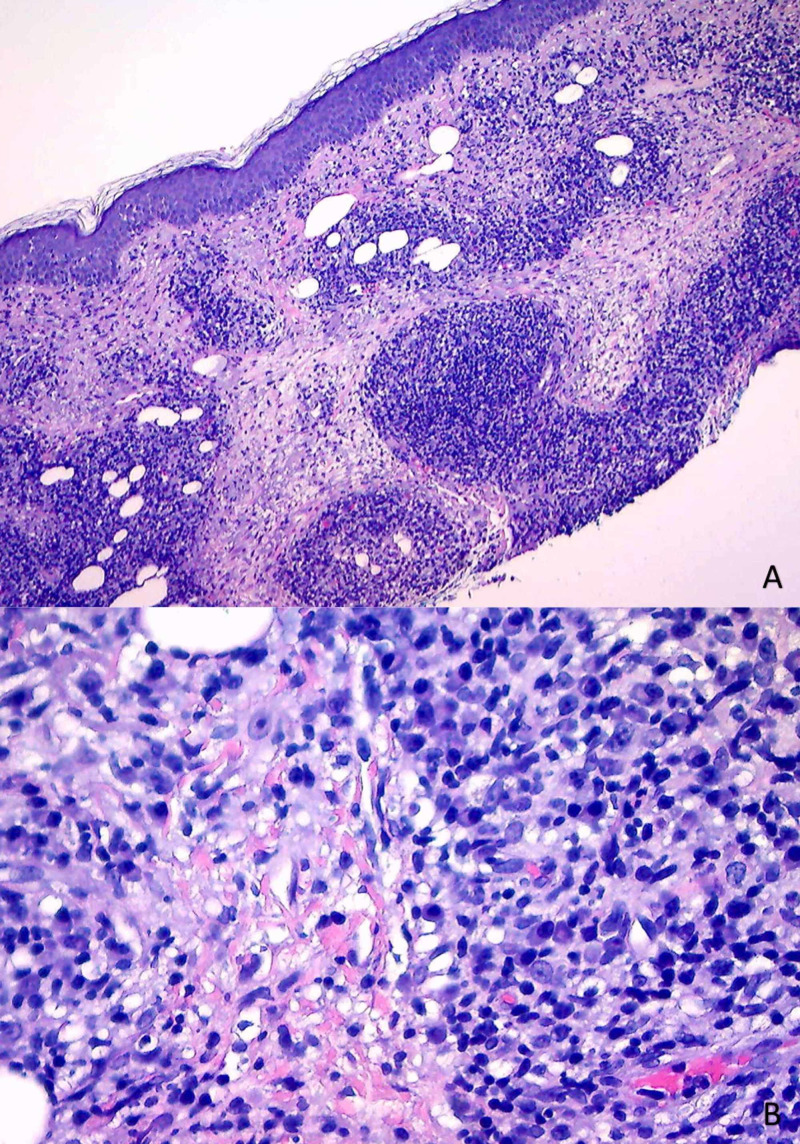
Histopathology of Anterior Neck Punch Biopsy Specimen (A) Hematoxylin and eosin (H&E) staining, magnification x10. Lichenoid dermatitis of the dermoepidermal junction, psoriasiform hyperplasia. (B) H&E staining, magnification x20. Extensive dermal plasma cell infiltrate.

Due to his past medical history of non-melanoma skin cancer and the possibility of an infectious process, a biopsy of the lesion was sent for further evaluation. Histopathology of the biopsy specimen revealed lichenoid dermatitis with psoriasiform hyperplasia. Additionally, an extensive infiltrate of plasma cells and lymphocytes was present within the tissue (Figure 1). At the time of the initial review, the histopathologic differential diagnosis included lichen planus, lupus, unusual arthropod reaction, and syphilis. The following ancillary procedures were performed: immunostain for *Treponema pallidum*, periodic acid-Schiff-diastase (PAS-D) stain, colloidal iron stain, immunostain for CD123, Fontana-Masson (FM), laminin, and CD30.

**Figure 2:**
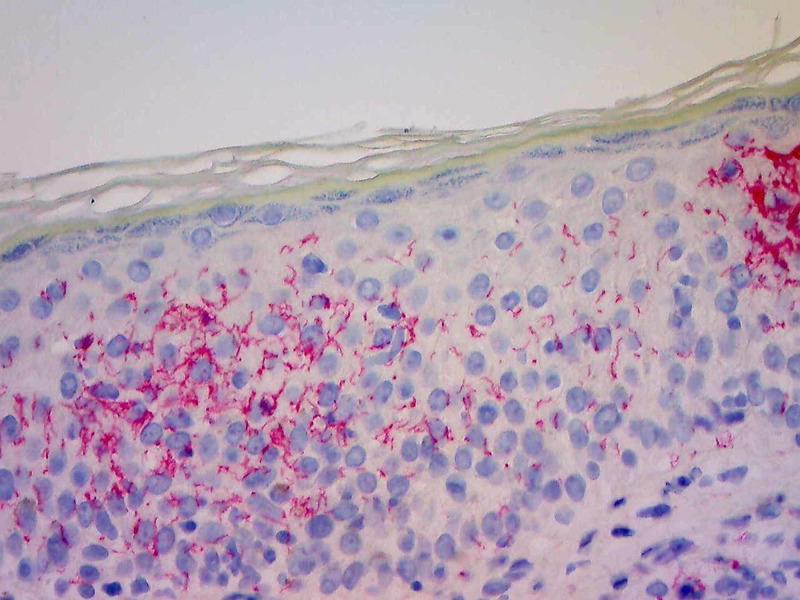
Immunohistochemical Staining with Spirochete Antibody of Anterior Neck Biopsy Numerous *Treponema* within the epidermis and dermis, magnification x20.

A diagnosis of syphilis was made based on positive immunohistochemistry (IHC) staining for *T. pallidum* coupled with characteristic histologic appearance (Figure 2). A differential of tertiary syphilis (evolving gumma) versus nodular secondary syphilis was raised. However, at the time of follow up two weeks later, the patient presented with the classic, symmetrical, reddish maculopapular rash involving the palms with a mild collarette scale. He again denied generalized symptoms of fever, malaise, change in cognition, and mood disturbances. No lymphadenopathy or mucous membrane involvement was appreciated on the secondary exam. A diagnosis of secondary syphilis was established, and the patient was referred to a local health clinic for treatment with intramuscular injections of benzathine penicillin. He was instructed to follow up with dermatology in three months to ensure the resolution of cutaneous symptoms.

## Discussion

Frequently, the primary chancre marking syphilitic infection can go unrecognized due to the absence of additional symptoms and swift resolution or an unusual primary location (within the anal canal or oral cavity) [7]. An asymptomatic period follows, during which spirochetes disseminate through the vasculature, leading to widespread systemic findings [2,7]. The typical cutaneous presentation of secondary syphilis has been well described, manifesting as a symmetric, nonpruritic, maculopapular rash ranging from red to brownish-copper in color. Lesions commonly cover the trunk and extremities and may extend to involve both the palmar and plantar surfaces [2,3,5]. Although these cutaneous findings are distinct, the clinical picture is not always characteristic [4,5]. Secondary syphilis is well known for physical variability and may present as symmetric macules, nodules, or papules or even as a solitary lesion [5].

Additionally, the association between syphilis and HIV coinfection has long been established. According to the Centers for Disease Control and Prevention, 41.6% of primary and secondary syphilis cases reported in 2018 among MSM with documented HIV status were HIV-positive [1]. It has been proposed that syphilitic infection facilitates HIV transmission, possibly due to increased disturbance of the genital mucosal barrier and epithelium [8,9,10]. Also, it is more likely for patients with HIV to present with atypical clinical picture syphilis [1,8,10]. Unusual dermatitis in an HIV-positive patient may lead to an incorrect diagnosis and a delay in imperative treatment [2,10]. In this case, the patient disclosed his HIV status at the time of the initial encounter. However, even if his HIV status were unknown, histology findings generated enough clinical suspicion for syphilis infection alone. His solitary crusted nodule preceded the characteristic exanthem of secondary syphilis by two weeks in the absence of regional lymphadenopathy and flu-like symptoms. Due to the location of his lesion, this reaction may have been correlated with an occult primary chancre within the oropharynx.

Interestingly, our patient did not present with multiple symmetric nodules as documented in several prior cases of nodular syphilis [5,6,11]. Rather, his single lesion on the anterior neck was mildly erythematous and crusted centrally, clinically resembling squamous cell carcinoma [12]. His past medical history of occupational sun exposure and squamous cell carcinoma further supported this differential. Also, our patient initially had no pathologic involvement of mucous membranes or regional lymph nodes, which are generally involved in secondary syphilis [5,7]. The presentation of nodular syphilis has mimicked lymphoproliferative disorders, sarcoidosis, psoriasis, fungal infections, and other various dermatoses [5,6,11]. This case discussion aims to add non-melanoma skin cancer to the expanding differential and stresses clinical follow up to distinguish tertiary and secondary variants of syphilis.

Histopathologic evaluation of secondary syphilis is often characterized by a heavy infiltrate of plasma cells and lymphocytes, with or without eosinophils, in lichenoid distribution at the dermoepidermal junction with psoriasiform hyperplasia of the epidermis [13]. Traditional silver-based staining techniques, including the Warthin-Starry stain, are less effective in the detection of *Treponema *spirochetes of secondary syphilitic lesions, further complicating diagnosis. For this reason, targeted IHC antibodies should be employed for detection when this distinctive pattern of plasma cell infiltration is present [13,14]. Our patient’s biopsy did not confirm the diagnosis of neoplasia as hypothesized. Rather, the combination of his infectious past medical history, histologic findings, and positivity with *T. pallidum* antibodies established the diagnosis of syphilis. Of note, presenting as a large nodule, a diagnosis of tertiary syphilis (gumma) was entertained but abandoned in favor of secondary nodular variant once the dermatitis appeared.

The detection of spirochetes in large numbers is possible in primary and early secondary cutaneous lesions. Further progression of the disease leads to fewer numbers of identifiable *T. pallidum* spirochetes, making a diagnosis more difficult [14]. Left untreated, secondary syphilis infection may resolve in weeks to months and enter the latent and tertiary disease stages [2,7]. Therefore, it is important to establish a diagnosis early in the clinical course of the disease. 

## Conclusions

The incidence rates of new cases of primary and secondary syphilis continue to rise in the United States. This increase necessitates the early diagnosis and treatment of syphilis in order to prevent disabling tertiary manifestations and substantial morbidity. Despite a heightened understanding of *T. pallidum* infection, the diagnosis of syphilis remains difficult. Secondary infection may present atypically and be easily mistaken for other dermatologic conditions, especially in patients with HIV coinfection. The authors of this case report intend to inform and caution physicians about the unusual presentation of nodular secondary syphilis. We suggest providers maintain an elevated suspicion of syphilis infection in high-risk patients, such as MSM, presenting with cutaneous lesions. Specifically, syphilis must remain on the differential diagnosis in HIV-positive patients who present with solitary, nonpruritic, crusted nodules resembling non-melanoma skin cancer, or histologically present with plasma-cell rich lichenoid and psoriasiform patterns.
